# Cutaneous anthrax associated with handling carcasses of animals that died suddenly of unknown cause: Arua District, Uganda, January 2015–August 2017

**DOI:** 10.1371/journal.pntd.0009645

**Published:** 2021-08-23

**Authors:** Freda Loy Aceng, Alex Riolexus Ario, Phoebe Hilda Alitubeera, Mukasa Matinda Neckyon, Daniel Kadobera, Musa Sekamatte, Denis Okethwangu, Lilian Bulage, Julie R. Harris, Willy Nguma, Deo Birungi Ndumu, Joshua Buule, Luke Nyakarahuka, Bao-Ping Zhu

**Affiliations:** 1 Uganda Public Health Fellowship Program, Kampala, Uganda; 2 Ministry of Health, Kampala, Uganda; 3 Arua District Local Government, Arua, Uganda; 4 Zoonotic Disease Coordination Office, Kampala, Uganda; 5 African Field Epidemiology Network, Kampala, Uganda; 6 Workforce and Institute Development Branch, Division of Global Health Protection, Center for Global Health, US Centers for Disease Control and Prevention, Atlanta, Georgia, United States of America; 7 Ministry of Agriculture, Animal Industries and Fisheries, Entebbe, Uganda; 8 Uganda Virus Research Institute, Entebbe, Uganda; 9 US Centers for Disease Control and Prevention, Kampala, Uganda; Oregon State University College of Veterinary Medicine, UNITED STATES

## Abstract

**Background:**

Anthrax is a zoonotic disease that can be transmitted to humans from infected animals. During May–June 2017, three persons with probable cutaneous anthrax were reported in Arua District, Uganda; one died. All had recently handled carcasses of livestock that died suddenly and a skin lesion from a deceased person tested positive by PCR for *Bacillus anthracis*. During July, a bull in the same community died suddenly and the blood sample tested positive by PCR for *Bacillus anthracis*. The aim of this investigation was to establish the scope of the problem, identify exposures associated with illness, and recommend evidence-based control measures.

**Methods:**

A probable case was defined as acute onset of a papulo-vesicular skin lesion subsequently forming an eschar in a resident of Arua District during January 2015–August 2017. A confirmed case was a probable case with a skin sample testing positive by polymerase chain reaction (PCR) for *B*. *anthracis*. Cases were identified by medical record review and active community search. In a case-control study, exposures between case-patients and frequency- and village-matched asymptomatic controls were compared. Key animal health staff were interviewed to learn about livestock deaths.

**Results:**

There were 68 case-patients (67 probable, 1 confirmed), and 2 deaths identified. Cases occurred throughout the three-year period, peaking during dry seasons. All cases occurred following sudden livestock deaths in the villages. Case-patients came from two neighboring sub-counties: Rigbo (attack rate (AR) = 21.9/10,000 population) and Rhino Camp (AR = 1.9/10,000). Males (AR = 24.9/10,000) were more affected than females (AR = 0.7/10,000). Persons aged 30–39 years (AR = 40.1/10,000 population) were most affected. Among all cases and 136 controls, skinning (OR_M-H_ = 5.0, 95%CI: 2.3–11), butchering (OR_M-H_ = 22, 95%CI: 5.5–89), and carrying the carcass of livestock that died suddenly (OR_M-H_ = 6.9, 95%CI: 3.0–16) were associated with illness.

**Conclusions:**

Exposure to carcasses of animals that died suddenly was a likely risk factor for cutaneous anthrax in Arua District during 2015–2017. The recommendations are investigation of anthrax burden in livestock, prevention of animal infections through vaccinations, safe disposal of the carcasses, public education on risk factors for infection and prompt treatment of illness following exposure to animals that died suddenly.

## Author summary

We are honored to submit an original research article entitled “Cutaneous anthrax associated with handling carcasses of animals that died suddenly of unknown cause: Arua District, Uganda, January 2015–August 2017”. Anthrax is a vaccine-preventable disease that can be transmitted to humans from infected animals. Our paper is based on 68 cutaneous anthrax case-patients and cases occurred throughout the three-year period, peaking during dry seasons in Arua District of Uganda. All cases occurred following sudden livestock deaths in the villages. Our findings are based on outbreak investigation which gives a better representation of the actual situation. Our article looks at the scope of the anthrax outbreak, exposures associated with illness, and evidence-based control measures. In this manuscript, we show that exposure to carcasses of animals that died suddenly was a risk factor for cutaneous anthrax in Arua District during 2015–2017. We believe the content and objectives of our work provide knowledge on epidemiology of cutaneous anthrax.

## Introduction

Anthrax is an acute zoonotic disease caused by the Gram-positive spore-forming bacteria *Bacillus anthracis* [1]. Most anthrax infections occur among animals, with occasional spread to humans [2]. Human infections often result from handling and / or consuming meat of infected livestock [1]. Humans may develop cutaneous, inhalational, gastrointestinal, and injection-associated infection [1,3]. Cutaneous anthrax accounts for 95% of human cases [1] and is characterized by itching and skin lesions starting 1–7 days after infection; the lesions eventually form depressed eschars [1].

Although infrequently documented, anthrax is likely common among animals in Uganda; the frequency of human infections is unclear. In 2004, an anthrax outbreak killed hundreds of animals, including hippo, zebras, elephants, and other wildlife in Queen Elizabeth National Park, in southwestern Uganda [4]. Reports of humans dying after eating meat from dead hippos were unconfirmed [4–6]. A 2010 report documented nearly 100 deaths from anthrax among hippos and buffalo in the same park [4], and in 2011, an anthrax outbreak was reported in the nearby Sheema District, affecting both humans and cattle [7].

On 7 May 2017, the Arua District Surveillance Officer was informed about two probable cutaneous anthrax human cases from Rigbo sub-county. The patients, siblings aged 6 and 12 years, presented to a health center with acute onset of skin lesions with depressed blackened centers and edema. Both children reported recently handling and consuming meat from a cow that died suddenly. Although the district health officers investigated, no samples were taken and the cases were unconfirmed. Both children recovered after antibiotic treatment, and no further cases occurred in the family.

On 5 June 2017, the Uganda Ministry of Health (MoH) was notified about the death of a 35-year-old resident of Rhino Camp sub-county (Mr. A, unconnected to the two children described above) who presented to a health facility on 4 June with restlessness, sweating, confusion, difficulty breathing, and an eschar on his back. He deteriorated quickly, and died on 5 June. According to his neighbor, he had carried and butchered meat from a cow that died suddenly on 11 May, and developed itching on the affected site on 14 May. No other animals fell ill on the farm; a neighbor also handled the meat, but did not fall ill. The affected site on Mr. A’s back turned into a papulo-vesicular lesion, which progressed into a black eschar. After initially trying herbal remedies to address the problem, the patient had sought medical care at a local clinic on 21 May. At the clinic, he was diagnosed with suspected Herpes Zoster infection and given intravenous ceftriaxone; the rationale for this treatment is not clear. A skin lesion sample from Mr. A on 5 June tested positive for anthrax by PCR at the Uganda Virus Research Institute. On 24 July, a bull residing in the same community as Mr. A died suddenly. Microscopic examination of a tissue sample from the bull showed Gram-positive rods, consistent with *B*. *anthracis*, and PCR of the tissue sample confirmed *B*. *anthracis*. During August 2017, a team from the Ministry of Health traveled to the area to establish the scope of the problem, identify exposures associated with transmission, and recommend evidence-based control measures.

## Methods

### Ethics statement

This investigation was in response to a public health emergency and was therefore determined to be non-research. The MoH through the office of the Director General of Health Services gave the directive and approval to investigate this outbreak. Additionally, the Centers for Disease Control and Prevention also determined that this activity was not human subjects’ research, and its primary intent was public health practice or a disease control activity (specifically, epidemic or endemic disease control activity). Verbal informed consent and assent were obtained from case-patients and/or their close relatives in case of death. Verbal informed consent were obtained in the local languages from case-patients ≥18 years and from caretakers of case-patients <18 years. Participants were informed that their participation was voluntary and their refusal would not result in any negative consequences. Unique identifiers were used to ensure confidentiality and the same was done for the controls.

### Study setting

The study area was Rigbo and Rhino Camp sub-counties which are located along the River Nile in Arua District. Arua District is located in northwestern Uganda and is bordered by the Democratic Republic of the Congo (DRC) on its western border, and is 80 km from the South Sudan Border to the north. More than 80% of the households are engaged in agriculture, and the district has multiple rich wildlife reserves [8,9]. Farming is the major economic activity in the district and livestock grazing is practiced along the river banks [10]. Interactions between wildlife-livestock-humans have been observed in the sub-counties and this is a key driver for anthrax outbreaks [11].

### Case definition

Early interviews with district authorities and local council chairpersons indicated that probable cutaneous anthrax cases had been occurring in the area since at least 2015. Therefore, we defined a probable case as acute onset of painless skin lesions with progression from papular to vesicular before forming an eschar (with a depressed blackened center) in a resident of Arua District from January 2015–August 2017. A confirmed case was a probable case with laboratory-confirmed *B*. *anthracis* by PCR of a skin lesion sample. The symptoms of anthrax considered were; skin lesions, black eschar, edema, itching, swollen lymph nodes, abdominal pain, diarrhea, abdominal swelling, vomiting, acute respiratory distress, chest pain, coughing blood, headache, altered mental status, cough, convulsions, neck pain/stiffness, difficult breathing, throat pain, photophobia, coma, anorexia, fever, fatigue and neck swelling [1].

### Sample analyses

The eschar was sampled by carefully lifting the eschar’s outer edge; a sterile swab was inserted, then slowly rotated for 2–3 seconds beneath the edge of the eschar.

The fresh swab sample was used for genomic and plasmid DNA extraction using commercially-available DNA Promega DNA IQ system (Promega corporation, USA) following manufacturer’s instructions.

Protective antigen (PA) primers and probe targeting pXO1 plasmid, capsule (Cap) primers and probe targeting pXO2 plasmid and RNase P primers [12] targeting the human ribonuclease P gene were used in three separate reactions tubes per sample. We used CDC-supplied primers PA-Forward: CGG ATC AAG TAT ATG GGA ATA TAG CAA, PA-Reverse: CCG GTT TAG TCG TTT CTA ATG GAT, PA-Probe: FAM-CTC GAA CTG GAG TGA AGT GTT ACC GCA AAT-BHQ1 and for Capsule; Cap-Forward: ACG TAT GGT GTT TCA AGA TTC ATG, Cap-Reverse: ATT TTC GTC TCA TTC TAC CTC ACC and Cap-Probe: FAM-CCA CGG AAT TCA AAA ATC TCA AAT GGC AT-BHQ1. For RNaseP: RP-Forward: AGA TTT GGA CCT GCG AGC G, RP-Reverse: GAG CGG CTG TCT CCA CAA GT and RP-Probe: FAM-TTC TGA CCT GAA GGC TCT GCG CG-BHQ1.

The PCR mixture (25μL total reaction volume) contained 12.5μl of Promega mastermix 2x M7505 (Promega corporation, WI USA) 0.1μM of each probe and 0.3μM of each primer and 5μl of DNA template. The RNaseP primers were used to confirm the presence of human DNA in the sample extracts. We used DNA from the pLepBaBp+, provided by CDC, as a positive control, and a non-template negative control (nuclease-free water). Each primer set was run in a separate PCR tube. Template DNA was initially denatured by heating at 95°C for 10 min, followed by 40 cycles of denaturation at 95°C for 15 sec, annealing and primer extension at 60°C for 1 min using the Stratagene MX3000P Real-time PCR system (Agilent Technologies USA, Santa Clara, California) [12]. Confirmed positive samples were those that amplified with both the pXO1 and pXO2 primers as well as the RNase P targets.

### Data collection

Interviews with the Arua District Health Officer (DHO) and District Veterinary Officer (DVO) suggested that Rhino Camp and Rigbo sub-counties were the source of most probable cases reported. As a result, the study was limited to these two sub-counties. Medical record reviews were conducted at the health centers in Rhino Camp and Rigbo to identify both previous and current probable anthrax cases. In addition, with the help of community health workers (CHW), the Assistant Animal Husbandry Officer, and health facility staff, active search for case-patients was conducted. The District Veterinary Officer (DVO), the Animal Husbandry Officer, and local council leaders were interviewed using key informant interviews about sudden livestock deaths to identify suspect animal anthrax cases. A suspected animal anthrax case was defined as an animal that died suddenly and had an epidemiologic link to a human case. This criterion excluded animal cases that had no epidemiological linkage to human cases. This was mostly based on the comparison with the probable human anthrax cases and their exposure to those animals prior to symptom onset.

The probable anthrax cases or their caregivers were interviewed with a standardized case investigation form to collect data on demographic characteristics, date of symptom onset, clinical characteristics, livestock ownership, recent livestock deaths, and cause of livestock deaths. In addition, further questions were asked about contact with livestock carcasses before their illness onset, including case-patient involvement in skinning/butchering a dead animal carcass, preparing and eating meat from a dead animal, carrying a dead animal and contact with live animals (milking). The probable anthrax case-patients were described on the basis of age, sex, place, time, and clinical characteristics. For livestock, data was collected on date and location of livestock deaths. Quantum Geographic Information System (QGIS version 2.8.1) [13] was used to construct maps to show the locations of case-patients and livestock deaths in Arua District. The latitude and longitude of individual case-patient residences were obtained.

### Analytical study

A frequency-matched case-control study was conducted using two randomly-selected neighborhood controls (independent of sex or age, but living in the same village as the case-patient at the time of diagnosis) for each case. A control was an individual with no history of probable anthrax during January 2015-August 2017. Case-patients and controls were interviewed about activities during the 14 days before the case-patient’s illness.

### Data analysis

Data were entered and analyzed using Epi-Info version 7.2.1.0 [14]. To account for the matched study design, the Mantel-Haenszel method [15] was used to estimate odds ratios (OR) and their 95% confidence intervals. Using population data of the district [16], attack rates (AR) were calculated by person (sex and age) and place (sub-county). The attack rates were calculated using the number of new cases of disease in population at risk divided by the number of persons in population at risk [17]. We conducted a 2D- hotspot analysis using R-statistical computing software to compute standard deviational ellipsoid from the average center of animal deaths to determine whether human anthrax cases were clustered around the animal deaths. We determined statistical significance of clustering based on 3 standard deviational ellipses (Z-score = 2.58) within the mean centre of animal deaths. We further examined the human cases clustering or dispersion from animal deaths over a range of 500 meters distance using Ripley’s K-function [18–20].

## Results

### Descriptive epidemiology

Overall, 67 probable and one confirmed case-patient were identified; two died [case-fatality rate (CFR) = 2.9%]. All the 68 case-patients (100%) presented with skin lesions and black eschars (S2 **Fig**).

Symptoms were variable and included both cutaneous and gastro-intestinal symptoms. Skin lesion refers to papules and /or vesicles in the skin due to cutaneous anthrax. The non-cutaneous clinical signs include; abdominal pain, diarrhea, abdominal swelling and vomiting.

The most common lesion sites were on the hand (83%) or back (4.4%). Among the cases, 63 (93%) reported that they had sought treatment for their illness; data on the specific treatments were not obtained. The epidemic curve showed noticeable peaks during March and April of 2016 and May 2017 (S3 **Fig**).

Exposure to the meat or carcass of livestock that had died suddenly was reported before illness onset for all case-patients; 5 were exposed to goats and 63 to cows.

The median age of the case-patients was 34 years (IQR: 26–42). Persons aged 30–39 years were most affected (AR: 40.1/10,000 population), and males (AR: 24.9/10,000 population) were more affected than females (AR: 0.7/10,000 population) p<0.0001(**Table 1).**

**Table 1:** Attack rates by age and sex during a cutaneous anthrax outbreak, (N = 68).

Sex	Frequency	Total Population (For the sub-counties)	Attack Rate/10,000
Male	66	26496	24.9
Female	2	28,704	0.7
**Age group (years)**		**(For the sub-counties)**	
<10	5	18,719	2.7
10–19	7	14,583	4.8
20–29	12	8,992	13.4
30–39	22	5,488	40.1
40–49	13	3,561	36.5
50+	9	3,857	23.3

The overall attack rate was 12.3/10,000 population across the two sub-counties. Rigbo sub-county was more affected (AR = 21.9/10,000) than Rhino Camp sub-county (AR = 1.9/10,000) p<0.0001(**S4 Fig**).

In general, the human case-patient residences appeared to be in close proximity (within a few meters) to the locations of the reported livestock deaths. All human anthrax cases were clustered within a distance of 3 standard deviational ellipses from the mean center of animal deaths (Rayleigh distribution) (**S5 Fig**).

Using the Riley’s K-function, the observed K-values was higher than the upper 95% confidence interval at a distance of 500 meters, therefore, spatial dispersion of human cases from animal deaths was not statistically significant (**S6 Fig**).

### Hypothesis generation findings

Of the 39 case-patients interviewed during hypothesis generation, 37 (95%) reported having butchered livestock that died suddenly (within hours of the animal’s apparent illness onset), 35 (90%) carried carcasses of livestock that died suddenly, 32 (82%) skinned dead livestock that died suddenly, and one (2.6%) slept on hides. Further investigation revealed that the six and 12-year old children whose illnesses were part of the initial reported cluster had also participated in handling and eating meat from the carcass of a cow that died suddenly. Their father, who had butchered the cow, was also a case-patient. We therefore hypothesized that butchering/carrying and skinning carcasses of livestock that died suddenly were likely risk factors for infection in this area. Although some case-patients reported that they had gastrointestinal illnesses as well, none were specific enough to classify as possible gastrointestinal anthrax.

### Case-control study findings

We enrolled 68 case-patients and 136 controls in the case-control study. Median age of case-patients was 34 years (IQR: 26–42) and for control-persons was 30 years (IQR: 24.5–41); 97% of case-patients, and 93% of control-persons were male (p = 0.25). All case-patients (100%) and most control-persons (99%) worked in contact with soil; 99% of case-patients and 96% of controls lived in homes with dirt floors (that is unfinished floors).

In the case-control study, 57 (84%) case-patients and 72 (53%) control-persons skinned animals that died suddenly (OR_M-H_ = 5.0, 95%CI: 2.3–11). Sixty-five (96%) case-patients and 76 (56%) control-persons butchered carcasses of animals that died suddenly (OR_M-H_ = 22, 95%CI: 5.5–89); 61 (90%) case-patients and 74 (54%) control-persons carried the carcass of an animal that died suddenly (OR_M-H_ = 6.9, 95%CI: 3.0–16) (**Table 2**).

**Table 2:** Distribution of exposure status among cases and controls during a cutaneous anthrax outbreak.

Exposure to dead livestock	Num. of participants		% exposed		Odds ratio (M-H) 95% CI	
	Cases (n = 68)	Controls (n = 136)	Cases	Controls		
Skinned	57	72	84	53	5.0	2.3–11
Butchered	65	76	96	56	22	5.5–89
Carried	61	74	90	54	6.9	3.0–16
Ate	66	111	97	66	16	4.0–66

Sixty-six (97%) case-patients and 82 (60%) control-persons reported that they had at least one of these exposures (OR_M-H_ = 22, 95% CI = 5–104). Among the 2 case-patients who did not have any of these exposures (children ages 1 and 3 years), both had reportedly eaten meat from a cow that had died suddenly.

### Key informant interviews

Arua District Veterinary Office recommends that when an animal dies outside the context of slaughter of a healthy animal, the community should notify the sub-county or district authorities, who decide whether or not to take a sample before safe burial of the carcass. However, interviews with key informants, including the District Veterinary Officer (DVO), the Animal Husbandry Officer, and local council leaders revealed that such livestock deaths are rarely notified to the authorities. Rather, residents frequently skinned and butchered the animals, so as not to waste the meat. The informants also indicated that thorough smoking of the meat before consumption is a typical practice.

## Discussion

Cutaneous anthrax cases in this region of Uganda during 2015–2017 were caused by skinning, butchering and carrying carcasses of infected animals that died suddenly. The 68 patients represent the largest collection of cutaneous anthrax cases ever reported in Uganda. Although a large number of probable cases were unconfirmed, the laboratory confirmation of anthrax from a dead bull and a human in the same community strongly suggests that probable cases were likely to be anthrax. Clinical signs of case-patients were also characteristic of cutaneous anthrax.

Handling carcasses of animals with suspected and confirmed anthrax has previously been established as a risk factor for cutaneous anthrax [21–26]. The location of the black eschars for most case-patients–on the hands and back—has also been observed in other studies demonstrating livestock-human transmission of anthrax [21], and is likely related to animal carcasses being carried over the shoulder. The community practice of smoking meat before consumption may have prevented cases of gastrointestinal anthrax. Whether or not this was protective is difficult to confirm; vegetative forms of *B*. *anthracis* are easily killed during normal cooking, but spores are much more resistant to adverse conditions and require heating to at least 100° C for 15 minutes for inactivation [27]. Although there were no gastrointestinal anthrax cases identified, illnesses might have been missed at health facilities or otherwise unreported based on the GI symptoms reported.

Adult males were most affected in this cluster of infections. This is unsurprising, as this group is most likely to be engaged in exposures to animal carcasses. However, two case-patients were under five years of age. For these case-patients, the only two not involved in direct contact with livestock carcasses, infection may have occurred when spores were brought into the house by a parent or other contact involved in skinning, carrying, butchering, or preparing the meat. This type of exposure has been described previously with pediatric anthrax cases [28] and may explain the illnesses among the youngest case-patients.

Although probable cases had been occurring throughout the study period, the Ministry of Health was only alerted to these cases in mid-2017. Anthrax testing is possible in Uganda, but it is infrequent because of incomplete reporting in veterinary surveillance systems, knowledge gaps among healthcare workers about anthrax and limited access to laboratory services. Because of the infrequent testing, both human and veterinary anthrax are almost certainly underreported in Uganda. Investigation of probable cases over a longer period (i.e., before 2015) or a prospective study could shed some light on the degree of underreporting.

The epidemic curve showed noticeable peaks in human cases for two years during March-May, which corresponds with the dry season in Uganda. During the dry season, livestock may be at increased risk of contracting anthrax from soil due to increased exposure to spores in soil/dust through close grazing and looser soil [29, 30]. In addition, stressors, such as poor nutrition or overheating and reduced resource availability, may lead to increased animal density in localized areas, such as along river banks, and increase the possibility of getting infected [15, 30]. For humans, food scarcity during the dry season may also force them to seek alternate sources of food, which could lead to their eating meat that they might otherwise avoid.

Cutaneous anthrax cases have been reported at the animal-human interface in several other regions globally including Bhutan, the Republic of Georgia, Kenya, and Tanzania [21,24,26,31]. In some cases, the outbreaks have been related to chronic food insecurity [32]. In a household survey in Zambia conducted after an anthrax outbreak linked to eating infected hippo meat, 23% of respondents mentioned that they would eat meat from dead animals again, despite the risk, due to food shortages [32]. Although we did not systematically collect data on this, several respondents reported to us anecdotally that they could not afford butchery-sourced meat.

Cutaneous anthrax is usually curable with prompt antibiotic therapy; approximately 10–20% of untreated patients die [33]. Although this therapy is readily available in Uganda, anecdotal evidence suggests that not all physicians are familiar with the presentation, and misdiagnosis can lead to delays in appropriate treatment. The late care-seeking behavior and subsequent difficulty with diagnosis of Mr. A likely contributed to his eventual death. Although most patients in this outbreak reported that they sought and received treatment, apart from Mr. A, we were unable to assess whether or not they were diagnosed and treated correctly.

The number of human cases and the time period over which they occurred also suggest more widespread livestock anthrax in this region, something that could potentially be prevented by animal vaccination. Effective herd immunity against anthrax in cattle requires at least 80% of the animals in an area to be vaccinated [34]. During an outbreak, all livestock in areas around the affected area should be vaccinated [34]. Despite anthrax being considered a priority zoonotic disease in Uganda [35], mass animal vaccinations are not currently done, primarily due to the low number of reported cases, nor are reactive vaccinations done during an outbreak. While farmers can choose to vaccinate their animals privately, many do not. There are unpublished draft national guidelines currently in place requiring livestock vaccination. In Zimbabwe, the government previously conducted national annual anthrax vaccination campaigns for livestock, but currently vaccinations are restricted to high-risk areas only. In spite of this, livestock producers in Zimbabwe are advised to ensure that farm animals are vaccinated against anthrax annually [34].

## Limitations

Because we conducted the investigation retrospectively, there is a possibility of recall bias including symptoms and date of onset, hence masking the actual anthrax prevalence in the district. In addition, it is likely that some cases were missed, which would have led to an underestimation of the magnitude of the outbreak. Therefore to minimize on missing out case-patients because of the long period, every interviewee was asked about others with similar symptoms and key informant interviews were conducted in order to pick out as much as possible. It is also possible that some were not true anthrax, leading to an overestimation. However all the case-patients had skin lesions and the black eschar which is pathognomonic for cutaneous anthrax therefore they were taken to be anthrax cases. Also the case definition included skin lesions and black eschar so that cutaneous anthrax case-patients were picked out. We had sparse data about the livestock deaths, preventing certainty that they were due to anthrax. However the case-patients mentioned that they had symptoms following contact with livestock that had died suddenly hence the linkage stated. In addition, we could not confirm the vast majority of cases, nor did we identify any cases of gastrointestinal anthrax. Although some patients had nonspecific gastrointestinal illnesses at the time of investigation, many were already taking antibiotic therapy, rendering sample testing ineffective. It is possible that gastrointestinal anthrax occurred and was missed.

### Conclusions and recommendations

The cutaneous anthrax cases occurring over a 3-year period in Uganda were associated with handling carcasses of animals that died suddenly. The key findings of this study presented proof of infected livestock carcasses within human vicinity and risky human behavior of handling and consuming infected livestock carcasses that caused a case-patient to succumb. The district set up a One Health structure (a collaboration between human and veterinary health departments, including a single laboratory that processes both human and veterinary samples) to facilitate epidemic preparedness and response as a result of this outbreak. The recommendations are investigation of anthrax burden in livestock, prevention of animal infections through vaccinations, safe disposal of the carcasses, public education on risk factors for infection and prompt treatment of illness following exposure to animals that died suddenly.

## Supporting information

S1 Data Final anthrax data set used. (XLSX)

S1 Fig Map showing study location. These maps show the location of the two sub-counties affected by the cutaneous anthrax outbreak. This map was created using QGIS 2.8.1 software.
(TIF)

S2 Fig Distribution of symptoms among 68 persons with probable and confirmed cutaneous anthrax. (TIF)

S3 Fig Epidemic curve showing symptom onset dates of persons with probable and confirmed cutaneous anthrax. (TIF)

S4 Fig Map showing the attack rate/10,000 population for the affected sub-counties during a cutaneous anthrax outbreak, N = 68. (TIF)

S5 Fig Spatial cluster Map showing the locations of livestock deaths and human cases of probable and confirmed anthrax. (TIF)

S6 Fig Ripley’s K Function for human and animal cases. (TIF)
