# Supplementary figures and images for: Cutaneous anthrax associated with handling carcasses of animals that died suddenly of unknown cause: Arua District, Uganda, January 2015–August 2017
Source: PLoS Negl Trop Dis. 2021 Aug 23;15(8):e0009645. doi: 10.1371/journal.pntd.0009645 (PMC8382178; doi:10.1371/journal.pntd.0009645)

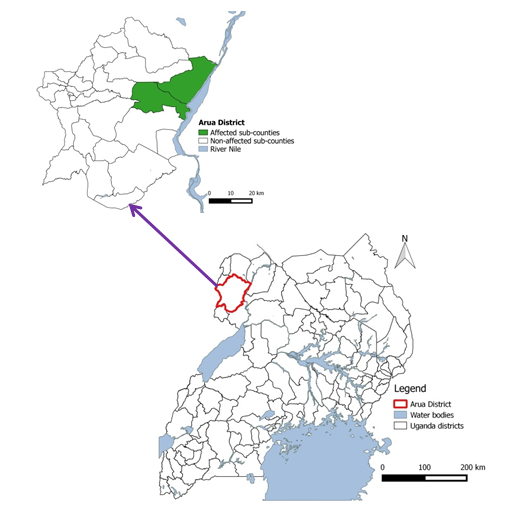

Supplement: S1 Fig — These maps show the location of the two sub-counties affected by the cutaneous anthrax outbreak. This map was created using QGIS 2.8.1 software. (TIF) [file pntd.0009645.s002.tif]

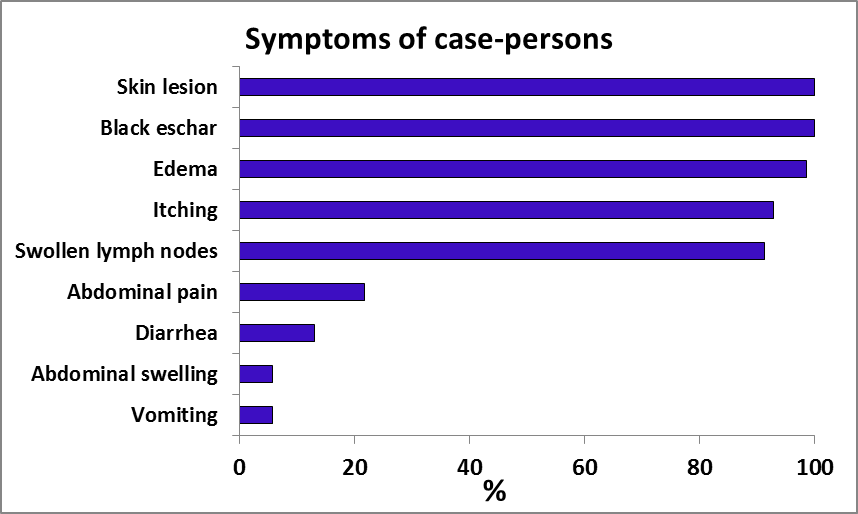

Supplement: S2 Fig — (TIF) [file pntd.0009645.s003.tif]

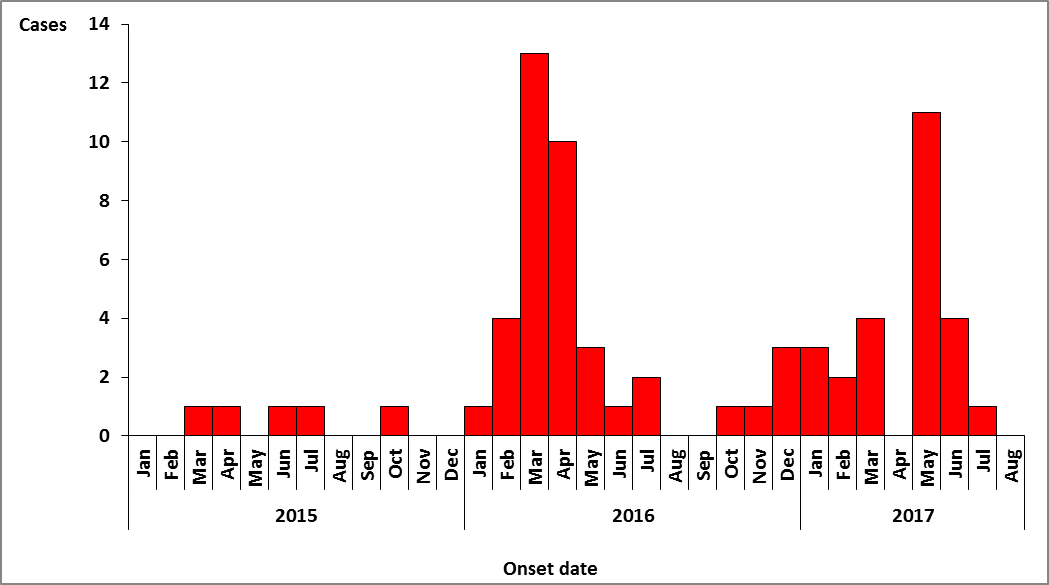

Supplement: S3 Fig — (TIF) [file pntd.0009645.s004.tif]

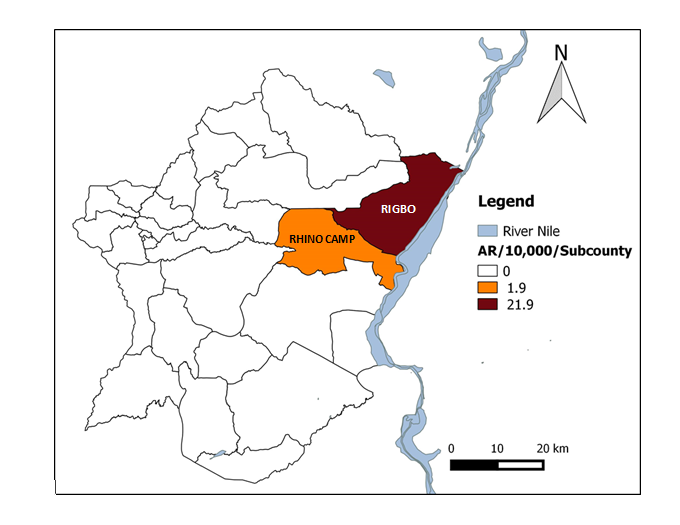

Supplement: S4 Fig — (TIF) [file pntd.0009645.s005.tif]

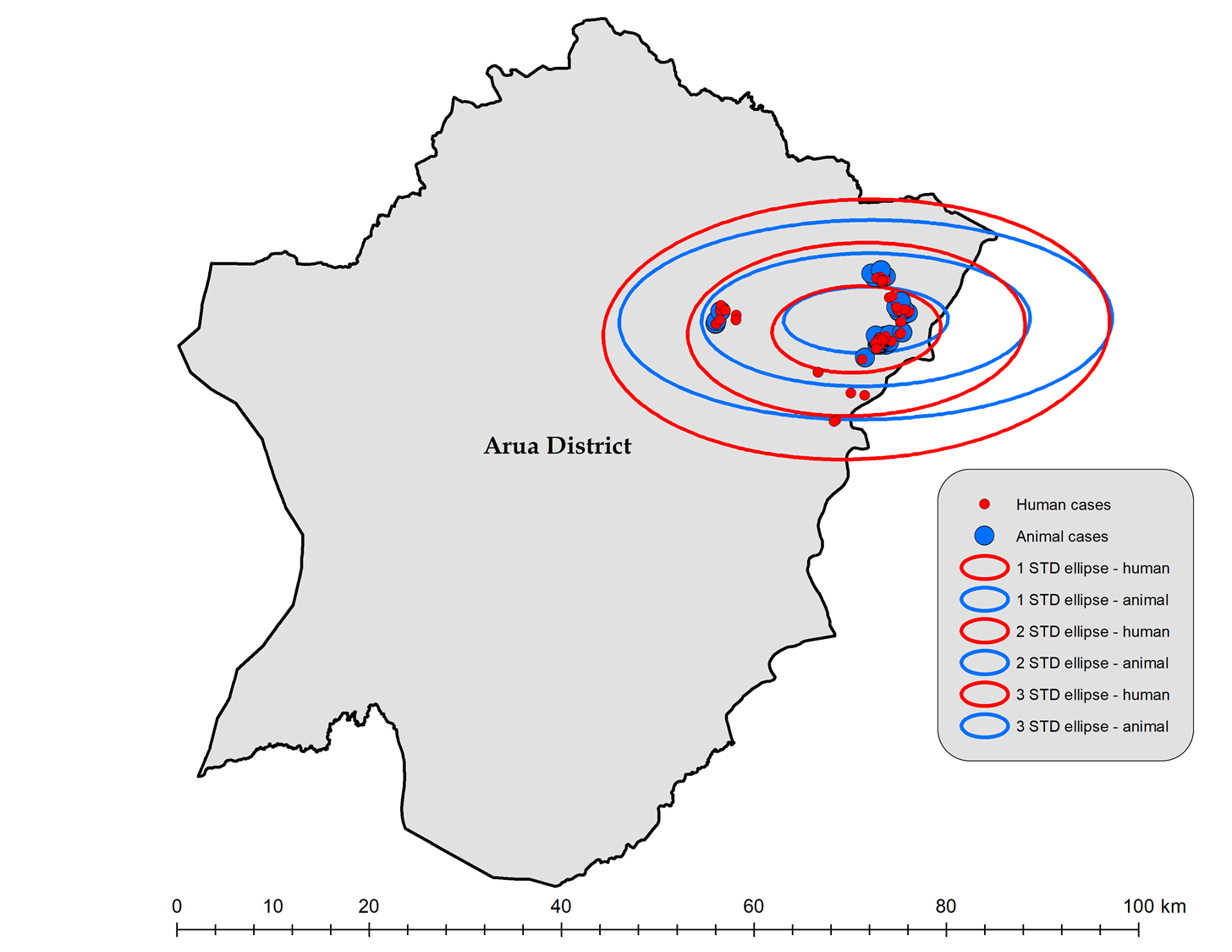

Supplement: S5 Fig — (TIF) [file pntd.0009645.s006.tif]

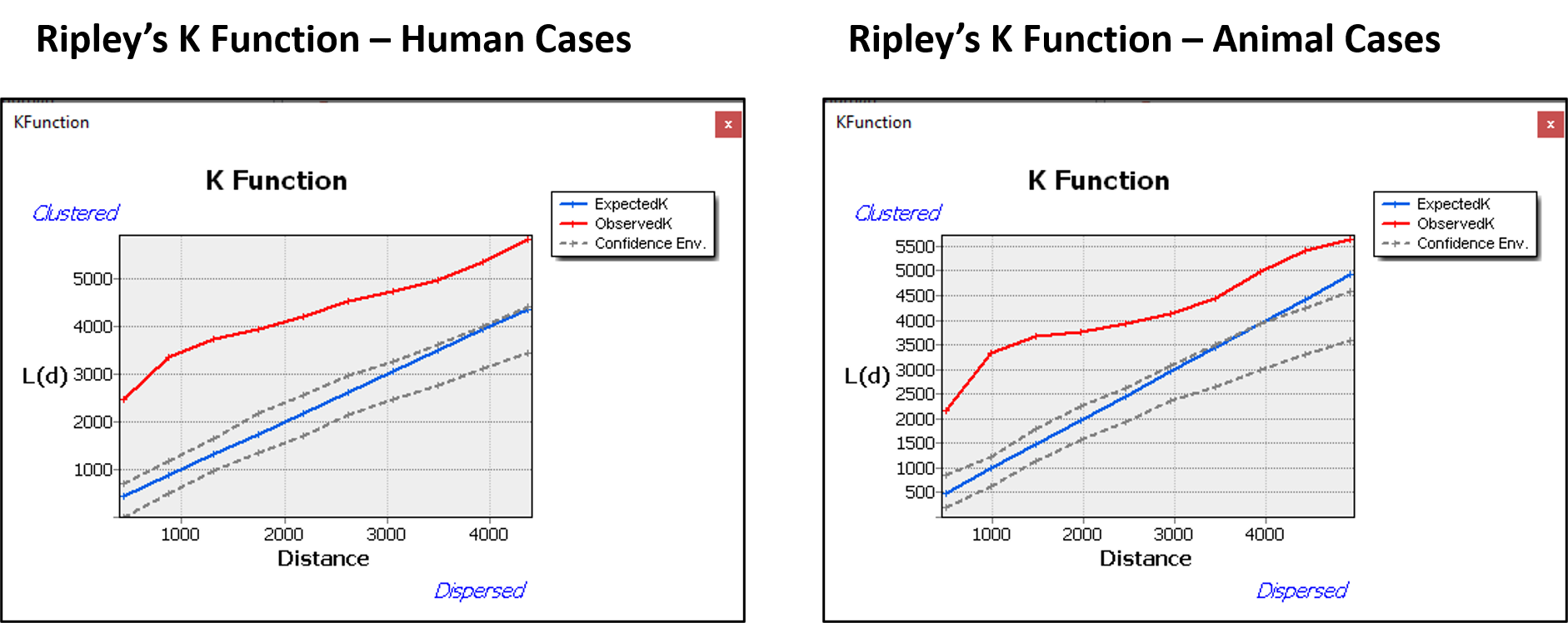

Supplement: S6 Fig — (TIF) [file pntd.0009645.s007.tif]
